# Heparin, Low Molecular Weight Heparin, and Non-Anticoagulant Derivatives for the Treatment of Inflammatory Lung Disease

**DOI:** 10.3390/ph16040584

**Published:** 2023-04-13

**Authors:** Janis Kay Shute

**Affiliations:** School of Pharmacy and Biomedical Sciences, University of Portsmouth, Portsmouth PO1 2UP, UK; jan.shute@port.ac.uk

**Keywords:** heparin, inflammation, coagulation, COVID-19, ALI, ARDS, cystic fibrosis, asthma, COPD

## Abstract

Unfractionated heparin has multiple pharmacological activities beyond anticoagulation. These anti-inflammatory, anti-microbial, and mucoactive activities are shared in part by low molecular weight and non-anticoagulant heparin derivatives. Anti-inflammatory activities include inhibition of chemokine activity and cytokine synthesis, inhibitory effects on the mechanisms of adhesion and diapedesis involved in neutrophil recruitment, inhibition of heparanase activity, inhibition of the proteases of the coagulation and complement cascades, inhibition of neutrophil elastase activity, neutralisation of toxic basic histones, and inhibition of HMGB1 activity. This review considers the potential for heparin and its derivatives to treat inflammatory lung disease, including COVID-19, ALI, ARDS, cystic fibrosis, asthma, and COPD via the inhaled route.

## 1. Introduction

Heparin is a mast-cell-derived biologic [1] for clinical use as an anticoagulant, isolated from porcine intestinal mucosa in Europe and the US, or from bovine intestinal mucosa in some South American countries such as Brazil and Argentina. In contrast to the widely used porcine mucosal heparin, the active pharmaceutical ingredient of bovine mucosal heparin has higher molecular weight [2] and a different structure [3], but may be substituted for porcine mucosal heparin as an anticoagulant [2]. However, porcine mucosal heparin is currently the only starting source for low molecular weight heparins, including the most widely used low molecular weight (LMW) heparin, enoxaparin. Unfractionated heparin (UFH) has multiple pharmacological activities beyond and independent of anticoagulation [4]. These anti-inflammatory, anti-microbial, and mucoactive activities are partly shared by LMW and non-anticoagulant heparin derivatives. This narrative review is based on searches of PubMed and Web of Science databases up to January 2023 for relevant references that evidence the potential for heparin and its derivatives to treat inflammatory lung disease, including COVID-19, acute lung injury (ALI), acute respiratory distress syndrome (ARDS), cystic fibrosis (CF), asthma, and chronic obstructive pulmonary disease (COPD) via the inhaled route.

## 2. Heparin Structure and Anticoagulant Activity

**Figure 1:**
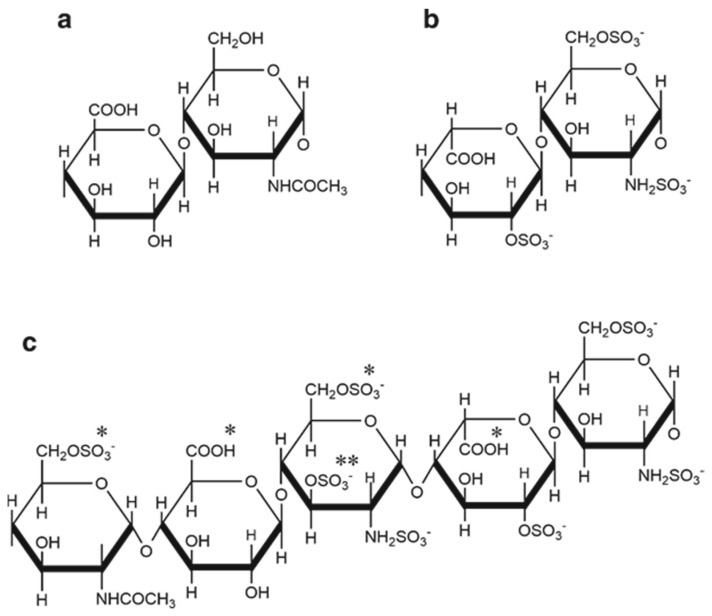
The main repeating disaccharide units found in the glycosaminoglycans heparan sulphate (**a**) and heparin (**b**). The polymers are composed of alternating hexuronate (β-d-glucuronate in HS (**a**) and α-L-iduronate in heparin (**b**)) and α-d-glucosamine residues, which are regularly 2-N and 6-*O* sulphated in heparin, joined by (1,4) glycosidic linkages. The anti-thrombin III high affinity binding pentasaccharide sequence found in pig mucosal heparin (**c**) contains essential substituents (*) and the unusual 3-*O*-sulphate substituent (**) Reprinted from ref. [1] (http://creativecommons.org/licenses/by/4.0/).

Heparin is a member of the glycosaminoglycan (GAG) family of linear polysaccharides, made up of repeating disaccharide units comprising a hexuronic acid (usually iduronic acid) and a glucosamine, which are N- and O-sulphated (Figure 1b) [1]. Heparin is synthesised by mast cells in mucosal tissues and is a more highly sulphated variant of the GAG heparan sulphate (Figure 1a) [1] that is found in association with core proteins in the extracellular matrix and at cell surfaces. Heparan sulphate retains low levels of N- and O-sulphation and heparin is therefore more negatively charged than heparan sulphate, and is one of the most negatively charged molecules in biology. In terms of charge, molecular size, and structure, heparin is synthesised as a highly heterogeneous molecule. The average molecular weight of commercially available UFH is 15,000 daltons (range 3–30,000 daltons). These properties affect the interaction of heparin with plasma proteins, including anti-thrombin III (AT-III), and other heparin-binding proteins [4].

The anticoagulant and antithrombotic mechanisms of heparin have been well described [5]. A specific high-affinity pentasaccharide sequence in heparin binds and activates AT-III by inducing a conformational change in the AT-III protein, leading to rapid inhibition of thrombin (FIIa) and factor Xa and the anticoagulant activity of heparin (Figure 1c) [1,4]. Due to the heterogeneous nature of the heparin molecule, the unique pentasaccharide sequence is found in only about one third of heparin chains [6]. Inhibition of thrombin requires simultaneous binding of both AT-III and thrombin to the heparin molecule, and is therefore dependent on the polysaccharide chain length of heparin, requiring a minimum of 18 saccharide units. Conversely, inhibition of FXa requires only the binding of the pentasaccharide to AT-III, without the requirement for FXa to also bind to heparin. LMW heparins therefore have greater anti-Xa activity than anti-IIa activity [6].

The LMW heparins are prepared from UFH by various chemical, physical, or enzymatic depolymerization techniques, and as a result are a diverse group of chemically distinct compounds [7,8].

LMW heparins have an average mass of less than 8000 daltons [7]. However, commercially available products in clinical use differ in terms of their average molecular weights and pharmacological properties, including their anti-FXa/anti-FIIa ratio (generally 1.5–4) and anti-coagulant properties, and are therefore not equivalent. As for UFH, LMW heparins are a mixture of polysaccharide chains, of which about one third have anti-coagulant activity [6].

The structure of bovine intestinal heparins is distinct from that of porcine intestinal heparins, and bovine heparins exhibit lower specific anticoagulant activity (units/mg). Anticoagulant activity correlates with the level of heparin 3-O-sulphation and the reduced content of N-sulpho, 3-O-sulpho glucosamine, the central and critical residue in the antithrombin III binding site, is responsible for the reduced anticoagulant activity of bovine intestinal heparin [9].

UFH and LMW heparins further synergistically increase total anticoagulant activity by inducing release of tissue factor pathway inhibitor α (TFPIα) from endothelial cells and GAG-binding sites in the vascular extracellular matrix [6,10]. TFPIα reduces procoagulant potential within healthy vasculature via inhibitory effects on TF-FVIIa (TF-factor VIIa) catalytic complex, FXa, and early forms of the prothrombinase complex (FVa-FXa) [10]. Conversely, TFPIβ is glycosylphosphatidyl inositol-anchored in the endothelial cell plasma membrane, does not bind to heparin, and does not inhibit prothrombinase.

The clinical indication for UFH and LMW heparins is the prophylaxis and treatment of venous thromboembolism (VTE), including deep vein thrombosis (DVT) and pulmonary embolism (PE). As a consequence of its large molecular size and high negative charge, UFH does not cross mucosal tissues, and in view of its rapid metabolism in the gut heparin is not orally available [11]. Administration of LMW heparin is therefore by injection subcutaneously and, since the bioavailability of subcutaneous UFH is lower than that of intravenous UFH, preferably intravenously for unfractionated heparin.For the treatment of inflammatory lung diseases, the inhaled route is an option and is discussed below.

Patients (1–5%) anti-coagulated with UFH are at risk of immune-mediated heparin-induced thrombocytopenia (HIT), although the risk is higher for unfractionated than LMW heparin, and for bovine compared to porcine heparin [12,13]. After heparin is administered, a complex can form between heparin and platelet factor 4 (PF4; CXCL4), a chemokine released by platelets. HIT occurs when a patient exposed to heparin forms IgG antibodies directed against the heparin–platelet factor 4 complex. These antibodies bind to Fc receptors on platelets and the heparin–PF4 complex, resulting in the activation of platelets and a high risk of subsequent arterial and venous thrombosis despite low platelet counts [13].

Beyond UFH and LMW heparins, a group of ultra-low molecular weight heparin mimetics with anticoagulant activity have been chemically synthesised [14]. Of these, fondaparinux (Arixtra), a methyl glycoside analogue of the pentasaccharide AT-III binding site of heparin, is approved for the treatment of venous thromboembolism. Fondaparinux is selective for FXa inhibition and binds weakly to platelet factor 4 and therefore, although controversial, fondaparinux may induce thrombocytopenia [15].

## 3. Heparin Binding Partners

A systematic investigation of heparin/heparan sulphate protein binding partners identified 435 human heparin binding proteins (HBPs) [16]. Functionally, the HBPs were associated with the biological processes of inflammation, immune responses, systems development, cell–cell signalling, and regulation of cell proliferation. Thus, HBPs are involved in pathways responsible for the control of key physiological processes, and their deregulation can lead to pathological conditions including inflammatory diseases.

Binding of HBPs to heparin depends on the overall molecular size, sequence, conformation, sulphation pattern, and negative charge of heparin and the presence of basic amino acids, lysine, and arginine in the HBP [17]. Thus, the binding of HBPs to heparin may depend on a highly specific sequence of monosaccharides, as in the case of AT-III, or may be non-specific and charge-dependent, as in the case of PF4 and protamine [18]. Protamine binding neutralises the AT-III dependent anticoagulant activity of heparin. It is the clinical antidote for overdose with UFH, and partially neutralises the anti-Xa activity of LMW heparin [6,19]. Protamine further reverses the anticoagulant activity of heparin, as heparin-released TFPIα redistributes out of the circulation on protamine administration. Protamine does not, however, bind to fondaparinux, for which there is no antidote [20].

## 4. Heparin Binding Proteins That Neutralise the Anticoagulant Activity of Heparin

PF4 released from the alpha granules of activated platelets binds to and neutralises the anticoagulant activity of heparin. Based on its affinity for heparin, PF4 was the first chemokine to be isolated and characterised [21]. The major physiological function of PF4 is to promote blood coagulation. This function is related to the neutralisation of endogenous heparin and the negatively charged heparan sulphate side chains of proteoglycans on the surface of platelets and endothelial cells, facilitating platelet aggregation and thrombus formation. Thus, PF4 may be an alternative to reverse heparin anticoagulation [6].

A number of basic proteins have similarly been shown to bind to heparin and neutralise its anticoagulant activity. These include further coagulation pathway factors (tissue factor, activated Factor VII, factor VIII), lactoferrin, histones, FGF-1, FGF-7, vitronectin, fibronectin, fibrinogen [22,23], and CXCL8 (IL-8) [24]. Longstaff et al. (2016) showed that the anticoagulant activity of both unfractionated and LMW heparin was inhibited by histones, and the effect was seen even in the presence of DNA [25], as occurs in neutrophil extracellular traps (NETS, see below). These HBPs therefore regulate the net anticoagulant activity of heparin and potentially contribute to the mechanism of heparin resistance [26].

## 5. The Anti-Inflammatory Effects of Heparin

**Figure 2:**
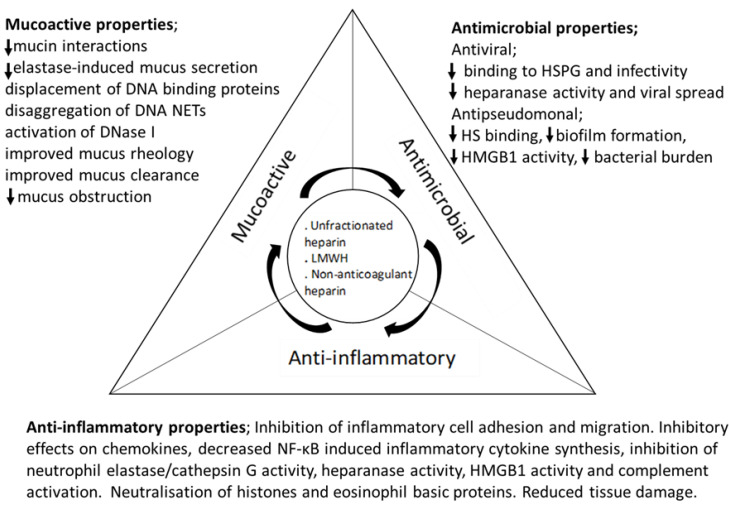
The anti-inflammatory, anti-microbial, and mucoactive pharmacological properties of heparin and its derivatives are proposed to reduce mucus obstruction of the inflamed airways, and the cycles of infection and inflammation that lead to tissue damage. NETs; neutrophil extracellular traps. HSPG; heparan sulphate proteoglycan. HS; heparan sulphate. HMGB1; high mobility group box 1. Downward arrows indicate a decrease in the described property.

Heparin has multiple anti-inflammatory, mucoactive, and anti-microbial pharmacological properties (Figure 2) with the potential to limit multiple aspects of the inflammatory response, including endothelial adhesion, migration and activation of leukocytes, and neutralisation of released tissue-damaging mediators (reviewed in [4]).

### 5.1. Inhibition of Chemokine Activity

The function of the pro-inflammatory chemoattractant cytokines, the chemokines [27], is largely determined by their binding to heparan sulphate (HS) side chains of the heparan sulphate proteoglycans (HSPG) of endothelial cells and the tissue matrix [28]. When chemokines are bound to HSPG, they form immobilised concentration gradients in which the chemokine is protected from proteolytic activity. In this form, HSPGs present the chemokines to specific leukocyte receptors and direct leukocyte trafficking *in vivo* [29].

HS is a highly heterogeneous molecule with one of the most diverse structures in biology, and UFH is a highly sulphated, more negatively charged variant of HS [30]. Soluble heparin can displace chemokines from the essential HSPG co-receptor binding sites, forming a complex that is unable to bind and activate chemokine-specific G-protein-coupled receptors, thereby inhibiting the directed migration of leukocytes [28]. Furthermore, soluble heparin and derivatives of heparin with reduced anticoagulant activity can interact with and displace IL-8 from surface-bound heparin *in vitro*, limiting its pro-inflammatory potential [31].

### 5.2. Inhibition of Leukocyte Adhesion to Endothelial Cells

In binding to L-selectin on leukocytes and P-selectin on endothelial cells, heparin interferes with the early HSPG-dependent selectin-mediated process of leukocyte rolling on the endothelium and the early sequestration of leukocytes during the inflammatory response [4]. These inhibitory effects are seen with therapeutically relevant concentrations of UFH, although potency is reduced 100-fold in the LMW heparins enoxaparin and dalteparin [32]. Thus, unlike UFH, LMW heparin was not an effective inhibitor of neutrophil adhesion to activated endothelial cells [33]. Additionally, pre-clinical studies indicated that UFH is a more potent local anti-inflammatory agent than LMW heparin (dalteparin sodium) or a selectively 2- and 3-O desulphated non-anticoagulant derivative of heparin [34].

Further, UFH binds the β2-integrin adhesion molecule MAC-1 [35] and inhibits the MAC-1 dependent firm adhesion of leucocytes to the endothelium [36], an essential step in transendothelial migration.

### 5.3. Inhibition of Neutrophil Elastase Activity

Unfractionated and LMW heparins inhibit neutrophil elastase release [33] and heparin has direct inhibitory effects on neutrophil elastase activity that correlate positively with the saccharide chain length and degree of sulphation [37]. A minimum chain length of at least 12–14 saccharide units was required for inhibition, after which the inhibitory activity increased with increasing chain length. Additionally, inhibitory activity is dependent upon the heparin sulphation pattern. Thus, although all N- and O-sulphate groups contribute to inhibition, 2-O-sulphate groups are less critical than either N- or 6-O-sulphate groups [37]. Similarly, high molecular weight N-acetylated and glycol-split derivatives of heparin with low anti-coagulant activity are potent neutrophil elastase inhibitors [31]. Furthermore, Rao et al. (2010) demonstrated that not only unfractionated heparin but also a low-anticoagulant derivative, 2-O, 3-O desulphated heparin (ODSH), are potent and effective inhibitors of neutrophil elastase and cathepsin G [38].

In addition to the direct inhibitory effect of heparin on elastase activity, as for the serine protease inhibitors (serpins) such as antithrombin that regulate protease activity in the coagulation cascade [39], heparin is also reported to enhance the activity of endogenous neutrophil elastase inhibitors, alpha1-anti trypsin [40] and secretory leukocyte protease inhibitor (SLPI) [41]. Inhaled heparin and/or non-anticoagulant derivatives therefore present therapeutic opportunities to limit neutrophil elastase activity in the airways and limit tissue damage.

### 5.4. Inhibition of High Mobility Group Box1 (HMGB1)

HMGB1 is a multi-functional nuclear protein, released extracellularly during cell death (necrosis, apoptosis, pyroptosis, and NETosis) where it has pro-inflammatory activity [42]. HMGB1 induces pro-inflammatory cytokine release by binding to the toll-like receptor 4 (TLR4) and receptor for advanced glycation end-product (RAGE) [43]. Heparin inhibits the inflammatory response induced by LPS and HMGB1 by inhibiting the binding of HMGB1 to receptors on the surface of macrophages [44]. Both UFH and ODSH interfere with HMGB1 binding to RAGE [38]. *In vivo*, intratracheal HMGB1 significantly increased total cells, neutrophils, total protein, and TNF-α concentration in mouse bronchoalveolar lavage (BAL) fluid 24 h after instillation. Simultaneous intratracheal instillation of low anticoagulant ODSH with HMGB-1 significantly decreased total cells, neutrophils, and TNF-α concentration in BAL fluid, indicating that ODSH can also inhibit proinflammatory HMGB1–RAGE interactions *in vivo* [38]. In addition, in line with its elastase-inhibitory capacity, ODSH was effective in inhibiting elastase-induced release of HMGB1 in the airway in a mouse model of intratracheal neutrophil elastase-induced airway inflammation, and blocked neutrophil elastase-stimulated HMGB1 release from murine macrophages *in vitro* [45].

### 5.5. Inhibition of Heparanase Activity

A role for heparanase activity in inflammatory cell trafficking promoting adhesion of leukocytes to endothelial cells and cell migration has been demonstrated in animal models *in vivo*, activity that was inhibited by UFH [46]. Heparin and non-anticoagulant derivatives are potent inhibitors of heparanase activity [47,48]. However, while heparin contains potential cleavage sites for heparanase, glycol-split non-anticoagulant derivatives such as roneparstat do not, and therefore may have a stable, prolonged, inhibitory effect [49].

### 5.6. Inhibition of Complement Activation

Complement activation in the lung generates pro-inflammatory complement-derived peptides, notably C3a and C5a, which recruit and activate phagocytes [50].

Heparin regulates multiple steps in the complement cascade. Heparin inhibits activity of the alternative, classical and terminal pathways of complement activation by regulating C1, C1 inhibitor, C4 binding protein, C3b, factor H, and S protein [51]. Although UFH preferentially inhibits the activity of the alternative versus the classical complement pathways [52], the inhibitory effect on the alternative pathway is reduced by depolymerisation [53]. However, high molecular weight heparin and derivatised (N-desulphated, N-acetylated) heparin with reduced anticoagulant activity both inhibit complement activation in human serum *in vivo* [51,54]. Low anti-coagulant 2-O,3-O-desulfated heparin (ODSH) also inhibits complement activation [38].

## 6. Mucoactive Effects of Heparin and Heparin Derivatives

Extracellular leukocyte DNA contributes to sputum elasticity and reduced cough clearance in inflamed CF airways [55]. UFH disaggregates DNA/actin bundles and activates endogenous and therapeutic DNase to reduce sputum elasticity and yield stress in CF sputum [56].

Neutrophil extracellular traps (NETs) are constructed of decondensed chromatin fibres with bound antimicrobial proteins, which may be granular or cytoplasmic, such as myeloperoxidase, neutrophil elastase, and α-defensins, and are part of the immune response to limit the spread of infection [57]. When DNA NETS are broken down, for example by DNase, the potential for the release of cytotoxic histones, neutrophil elastase, and other cationic proteins encrypted by the DNA [57] may be mitigated by the ability of heparin to bind and neutralise these basic proteins (see above).

The ability of UFH to decondense nuclear chromatin depends on the sulphation pattern of the molecule. UFH, O-desulphated heparin and N-desulphated-N-acetylated heparin were similarly active, while N-desulphated was less active and O/N-desulphated-N-acetylated heparin was completely inactive. However, the decondensing ability of heparin was not affected over the molecular weight range 3000–18,000 Da [58]. The displacement of basic proteins from chromatin by binding to heparin leaves naked DNA, a more accessible substrate for DNase I. Heparin facilitates the degradation of NETs by binding to and disassociating histones from the DNA backbone [59]. Other studies have reported that the LMW heparin, enoxaparin, is able to prevent the formation of PMA-induced NETs [60]. However, in a note of caution, heparin has been reported to induce elastase and reactive oxygen species (ROS)-dependent NET formation, although low molecular weight heparin, fondaparinux and heparan sulphate were less effective in inducing NET formation [61].

In addition, electrostatic mucin interactions and viscosity are increased by low pH in the airway surface liquid, as observed in CF, asthma, COPD, and ARDS, and these interactions are also reversed by heparin [62,63]. Furthermore, heparin inhibits the mucus secretory activity of neutrophil elastase and cathepsin G on airway submucosal glands [64]. The mucoactive effects of heparin are therefore proposed to reduce airflow obstruction in inflammatory airway diseases via effects on mucus rheology and inhibitory effects on mucus secretion.

## 7. Heparin as a Systemic Anticoagulant in COVID-19

Thrombosis is a frequent complication of COVID-19, especially venous thromboembolism, but also arterial thrombosis following infection with the severe acute respiratory syndrome (SARS)-CoV-2 virus [65]. A recent systematic review reported PE in a median 22.1% of hospitalised non-ICU patients and 44.9% of ICU patients, detected by CT pulmonary angiogram, otherwise the conditions remain under-diagnosed [66]. Segmental arteries were the most common location for PE. A high incidence of pulmonary thromboembolic events was reported at autopsy, including PE (6–23%), thrombosis in situ due to local thrombo-inflammatory disease (43–100%), and microthrombi in small arteries, arterioles, and alveolar capillaries (45–91%), as well as endothelial damage and necrosis.

The immune response to infection results in activation of coagulation pathways, leading to overproduction of proinflammatory cytokines and multiorgan injury [67]. Hypercoagulability in COVID-19 is associated with increased fibrinogen and D-dimer levels in the circulation [65]. However, pro-coagulant changes are not only observed intravascularly. Fibrin formation in the intra-alveolar space reflects localised microthrombi and endothelial damage in the pulmonary microcirculation, leading to plasma exudation, tissue factor-mediated thrombin generation, and the development of fibrinous hyaline membranes, a feature of the inflammatory response in ARDS [68].

In view of the coagulopathy associated with COVID-19, studies have investigated the beneficial, or otherwise, effects of anticoagulation with systemic heparin therapy. In an early retrospective study, Tang et al. (2020) reported that prophylactic treatment with LMW heparin for 7 days appeared to be associated with better prognosis in severe COVID-19 patients with coagulopathy and elevated D-dimer levels [69].

Subsequently, there have been more than 44 randomised controlled clinical trials of intermediate and therapeutic doses of LMW heparin and UFH [70]. The International Society on Thrombosis and Haemostasis (ISTH) published updated guidelines in 2021 for the prophylactic, intermediate, and therapeutic dosing of systemic heparin in patients with COVID-19 [71]. In non-critically ill patients not requiring mechanical ventilation or organ support other than low-flow supplemental oxygen, a low (prophylactic) dose of LMW heparin or UFH is recommended to reduce risk of thromboembolism and possibly death. In select non-critically ill patients hospitalized for COVID-19, and not at risk of bleeding, therapeutic dose LMW heparin or unfractionated heparin is beneficial in preference to prophylactic or intermediate dose heparin for reducing risk of thromboembolism and end organ failure. In critically ill patients, prophylactic doses of heparin but not intermediate and therapeutic doses are recommended. However, the risk/clinical benefit of higher doses is uncertain [72,73,74,75,76], and heparin resistance was reported to be high in COVID-19 patients in the ICU [77].

Despite the widespread use of prophylactic systemic anticoagulation with heparin, anticoagulation failure with thrombotic events on prophylactic heparin treatment [78] may be related to the mechanisms of heparin resistance described above, or extreme hypercoagulability in COVID-19 due to mechanisms not affected by heparin. Additionally, HIT remains a risk of treatment with UFH in COVID-19 patients [79].

## 8. Inhaled Heparin as an Anticoagulant, Anti-Viral, Anti-Inflammatory, and Mucoactive Therapy in COVID-19

Autopsies have revealed that SARS-CoV-2 can be found in nearly all human tissues [80]. However, lung disease is the leading cause of death, and the use of nebulised inhaled UFH to limit coagulopathy in the pulmonary compartment in COVID-19-induced ARDS has been proposed [81]. This approach may increase the efficacy of the treatment, allowing higher doses while avoiding complications associated with systemic administration. The multiple pharmacological actions of heparin [4], including anti-viral, anti-inflammatory, and mucolytic activity, are further proposed to provide clinical benefit across the course of the disease [81,82].

A retrospective study of inhaled UFH in 98 patients with COVID-19 demonstrated the safety of this approach [83]. Inhaled heparin at doses up to 100,000 U daily for 6 days did not induce clinically relevant systemic anticoagulation [83]. Others reportedthat inhaled heparin at 100,000 U daily (25,000 U q.i.d) for 10 days is safe and attenuated lung injury [84], and 10,000 U daily for 7 days reduced the number of admission days and need for mechanical ventilation [85] in patients with ARDS. These results further support the rationale for studies of inhaled heparin in COVID-19 patients. Consequently, a number of prospective randomised clinical trials of inhaled nebulised UFH are recruiting amongst COVID-19 patients, both those requiring mechanical ventilation and those not [86,87,88].

In a recent clinical trial, 10-day treatment with 4000 U LMW heparin (enoxaparin) inhaled twice a day improved hypoxaemia when delivered via soft-mist inhaler in patients with COVID-19, indicative of effects on pulmonary microvascular thrombosis [89].

Multiple anti-viral, anti-inflammatory, and mucolytic effects of inhaled heparin have been described, providing the rationale for inhaled heparin as a therapy in COVID-19 and other obstructive inflammatory airway diseases.

### 8.1. The Anti-Viral Properties of Heparin in COVID-19

Heparan sulphate proteoglycans (HSPG) on cell surfaces play an important role in attachment and infection of host cells by many viruses [90], including the SARS-CoV-2 virus [91]. Clausen et al. [91] showed that heparan sulphate interacts with the receptor-binding domain of the SARS-CoV-2 spike protein, shifting the spike structure to an open conformation to facilitate ACE2 receptor binding.

It was recently demonstrated [92] that UFH binds to the SARS-CoV-2 spike protein in charge-dependent interactions and blocks SARS-CoV-2 infectivity by three mechanisms: (1) allosterically hindering binding to the host cell ACE-2 receptor; (2) directly competing with binding to host HSPG coreceptors; and (3) by preventing spike cleavage at the site between the S1 and S2 subunits by the protease furin, hindering activation of the conformation of the spike glycoprotein which is required for fusion with the cell membrane and internalisation of the virus. UFH inhibits SARS-CoV-2 cell infectivity at therapeutically relevant concentrations and is 150-fold more potent than LMW heparin [93]. These anti-viral effects of heparin are independent of anticoagulant properties and are also observed with non-anticoagulant derivatives of heparin [93].

In addition to blocking viral access to heparan sulphate binding sites on cells, UFH, LMW heparin, and non-anticoagulant heparin (roneparstat) are potent heparanase inhibitors [4,94]. Heparanase enzyme activity degrades cell-surface heparan sulphate chains, facilitating the release of the virus after replication, increasing spread and transmission, and driving viral pathogenesis [95]. Heparin-mediated inhibition of heparanase activity was recently shown to limit SARS-CoV-2 infectivity *in vitro* [96], and when inhaled may similarly limit viral spread and the development of COVID-19.

Further recent studies have focussed on the antiviral activity of endogenous AT-III. The binding of anticoagulant heparins including fondaparinux to AT-III enhances the ability of AT-III to inhibit the TMPRSS2 membrane-bound serine protease on endothelial and epithelial cells that primes the spike protein for viral fusion, increasing the antiviral activity of endogenous AT-III [97]. The physiological concentration of AT-III in plasma may be sufficient to limit SARS-CoV-2 infection and replication, although the concentration in airway fluids may not be high enough [98] to protect against the acquisition of SARS-CoV-2 in the airways.

The ability of heparin to block viral infection of airway cells is likely to suppress subsequent events in the pathogenesis of COVID-19, although additional anti-inflammatory properties (see below) may be important.

### 8.2. Inflammation and the Potential Anti-Inflammatory Effects of Heparin and Derivatives in COVID-19

Initial infection with SARS-CoV-2 stimulates a pulmonary and systemic inflammatory response which varies greatly between individuals. In the majority of cases, there are no or only mild symptoms and fewer than 3% of infected individuals are hospitalised [99]. However, most patients who develop alveolar inflammation and pneumonia are hospitalised. Of these, nearly a third need to be admitted to the ICU [100] as the inflammatory response leads to tissue damage which can evolve into ARDS and hypoxemia that may require mechanical ventilation. In a meta-analysis of 46,959 confirmed cases of COVID-19-induced pneumonia, the incidence of ARDS was 28.8%, the incidence of multiple organ dysfunction syndrome was 8.5%, and the fatality rate was 6.8%, as lung function deteriorated rapidly [100].

The chemokine family of leukocyte chemoattractants play an important role in the inflammatory response and in the development of ARDS in COVID-19 (reviewed in [101]). The chemokines CCL2 (MCP-1), CXCL10 (IP10), and IL-8 correlated best with disease progression. Critically ill patients have been characterised by a neutrophil-dominant alveolar phenotype and a high pulmonary-to-blood concentration gradient of the neutrophil chemoattractant chemokine IL-8 [102]. Those patients with poor outcomes (either the patient needed organ support or was deceased 28 days following ICU admission) were associated with higher levels of IL-8 in bronchial lavage fluid and blood than those with good outcomes [102]. Other researchers [103] reported that, in patients with severe COVID-19 and ARDS, high levels of the monocyte chemoattractant MCP-1 drive recruitment of monocytes from the circulation and lead to a preponderance of hyperinflammatory macrophages in airway fluids and lung tissue. These tissue macrophages are the source of multiple inflammatory cytokines involved in monocyte (MCP-1), CD4+ lymphocyte (CCL3 (MIP-1α)), CD8+ lymphocyte (CCL4 (MIP-1β)) [104], and neutrophil (CXCL5 (ENA-78) and IL-8) recruitment [27]. Overall, elevated concentrations of chemokines dominate the hyperinflammatory cytokines detected in bronchial lavage fluid in severe COVID-19 [105].

Distinct serum cytokine profiles are observed in association with COVID-19 severity, with the most severe patients having elevated levels of TNF-α, IL-6, IL-8, and IL-10 [106]. Elevated blood levels of IL-6 are reported in many, but not all, studies [105,107,108,109]. A central role for plasma IL-6 in disease progression and severity was proposed, with a close association between inflammatory and thrombotic events [108]. IL-6 is also elevated in the bronchoalveolar lavage (BAL) fluid of patients with severe COVID-19 compared with healthy controls [105] and in severe compared to moderate disease [110]. This latter study demonstrated that elevated levels of both IL-8 and IL-6 were associated with neutrophilic inflammation and clinical outcomes, and both IL-8 [111] and IL-6 are considered targets for immunological therapies.

The anti-inflammatory effects of systemic and nebulised pulmonary heparin are proposed to target and inhibit not only the activity of the chemokines, including IL-8 and MCP-1, through interference with HSPG-dependent activity, as described above, but also through mechanisms that limit the expression and bioactivity of IL-6 [112]. Reduced expression of IL-6 may relate to reduced NF-kB activation in endothelial cells exposed to UFH [112]. In a retrospective study, LMW heparin was shown selectively to reduce plasma IL-6 concentrations, but not concentrations of IL-2, TNF-α, IL-4, IL-10 or IFN-γ in patients with COVID-19 [113]. However, Buijsers et al. (2020) reported no effect of prophylactic LMW heparin (dalteparin) on plasma IL-6 levels in moderately ill COVID-19 patients [114].

Receptor-mediated IL-6 activity is also regulated by HSPG-binding on cell surfaces and is displaced by UFH [115], limiting the bioactivity of IL-6 and interfering with receptor binding [112]. Selectively modified heparin derivatives (N-desulphated, 6-O-desulphated, and 2-O-desulphated) were less effective or less potent [115].

IL-6 is a pleiotropic cytokine. High levels of IL-6 rapidly released following infection activate the coagulation pathway [116], increase expression of acute phase proteins including fibrinogen, and increase expression of antifibrinolytic PAI-1 [117]. IL-6 stimulates the development of pro-inflammatory Th17 cells while reducing regulatory T cell numbers. IL-6 increases VEGF synthesis, angiogenesis, and vascular permeability. Furthermore, IL-6 increases endothelial expression of adhesion molecules and molecules that regulate inflammatory cell migration, such as MCP-1, a feature of COVID-19 [103].

In addition to the proposed inhibitory effects of heparin on inflammatory cell recruitment, heparin may also inhibit the effects of potent mediators of tissue damage released from activated leukocytes, including heparanase, histones, and neutrophil elastase.

Disruption of endothelial function is widely associated with thrombosis, and also with plasma leakage leading to pulmonary oedema and ARDS in COVID-19 [118]. Heparanase-mediated damage to the endothelial glycocalyx releases pro-inflammatory HS fragments [114] and damages the natural anticoagulant properties of the endothelial cell surface, effects that can be reversed with LMW heparin [119]. Plasma heparanase activity is significantly increased in the circulation in patients with COVID-19 [114,119] and is associated with disease severity, being highest in ICU patients receiving mechanical ventilation [114]. The use of prophylactic LMW heparin (dalteparin) reduced heparanase activity in moderately ill patients, but not those in ICU [114].

### 8.3. Effects of Heparin on DNA NETS, Histones, Neutrophil Elastase, Alveolar Damage and Fluid Exudation in COVID-19

Neutrophil extracellular traps (NETS) are composed of cell-free DNA with bound basic proteins including histones, myeloperoxidase, and neutrophil elastase [120], and are part of host immune responses to trap and limit the spread of pathogens.

A wide array of pathogens trigger DNA NET formation, including viruses, and recent evidence suggests that viable SARS-CoV-2 can dose-dependently stimulate human neutrophils to release NETs [121]. Other triggers for NET formation include activated platelets and complement activation [120]. Once formed, NETs stimulate thrombo-inflammatory responses in COVID-19 [122,123].

DNA NETS have been reported in the sera of patients with COVID-19 [124,125]. Neutrophil infiltration of the lung has been described at autopsy in fatal COVID-19 [123,126]. Furthermore, abundant neutrophils undergoing NETosis were observed in lung interstitial tissue and surrounding bronchiolar epithelium in association with IL-8 expression, which was proposed to be a causative factor [125,126]. Neutrophils and NETs were also detected in pulmonary thrombi from patients with fatal COVID-19, linking inflammation and thrombosis [126].

The tissue-damaging properties of NETs may contribute to respiratory failure. There is growing evidence that histones are the cytotoxic components in NETs that harm the endothelium and epithelium and are key mediators of lung injury and disease progression in COVID-19 [127] and in ARDS [128]. Heparin and antithrombin affinity-depleted, non-anticoagulant heparin neutralise the cytotoxicity of histones [129]. Heparin inhibits histone-induced alveolar macrophage pyroptosis in ARDS [128] and attenuates histone-induced inflammatory cytokine, including IL-6 and IL-8, responses in whole blood [130]. Thus, the heparin-mediated inhibition of histone and IL-8 function is reciprocal, since these HBPs also inhibit the anticoagulant activity of heparin (see above).

Plasma levels of neutrophil elastase, a potent elastolytic tissue-damaging enzyme found associated with NETs, were higher in patients with COVID-19 who died in hospital than those who survived and were discharged [131]. A protease–anti-protease imbalance, with active neutrophil elastase and no alpha1-antitrypsin, has been reported in the airways of SARS-CoV-2-ARDS patients [132]. This imbalance is therefore a potential target for inhaled anti-protease therapy, using UFH or non-anticoagulant derivatives as potent inhibitors of neutrophil elastase activity (see above).

Finally, extracellular high-mobility group 1 (HMGB1) acting on toll-like receptors (TLRs) and receptor for advanced glycation end-product (RAGE) activates release of pro-inflammatory cytokines from monocytes and macrophages. HMGB1 is found at high levels in the serum in COVID-19 patients. It is associated with tissue damage, and linked with disease severity, development of a cytokine storm, ALI, and ARDS (reviewed in [43]). Heparin is an anti-HMGB1 agent with a wide range of anti-inflammatory effects that may be further mediated via inhibition of HMGB1 activity and neutrophil elastase-induced release of HMGB1 in the airways.

## 9. Inhaled Heparin for ARDS and ALI

The outcomes of clinical trials investigating the safety and efficacy of inhaled heparin in patients with ARDS and ALI were recently reviewed [133].

Pulmonary coagulopathy is a characteristic of ARDS and lung injury [68] including ventilator-induced lung injury [134]. In mechanically ventilated patients with acute lung injury (ALI), four doses of UFH were delivered by nebulisation to four groups of four patients over 2 days. The first group was administered 25,000 U b.i.d. every 12 h, the second group received 50,000 U b.i.d. every 12 h, the third group received 100,000 U b.i.d. every 12 h, and the fourth group received 100,000 U q.i.d every 6 h, in addition to prophylactic systemic heparin in 14/16 cases. No adverse events were observed and the maximum nominal daily dose (400,000 U) significantly reduced pulmonary coagulation, with non-significant effects on systemic coagulation [135,136]. In a single-centre study, nebulised heparin at doses up to 150,000 U daily for up to 14 days was associated with significantly fewer days of mechanical ventilation in critically ill patients expected to require prolonged mechanical ventilation. The study showed no adverse events associated with inhaled heparin and only non-clinically significant effects on systemic coagulation [137]. However, a separate meta-analysis of patient data concluded that the evidence for the benefits of nebulised heparin was not convincing [138]. Subsequently, a multi-centre phase 3 study with mechanically ventilated patients with or at risk of ARDS demonstrated that nebulised unfractionated heparin (100,000 U daily, 25,000 U every 6 h, for 10 days) was well tolerated, without adverse effects, and attenuated lung injury with fewer cases of ARDS in at-risk patients [84].

The effect of inhalation of lower doses of nebulised heparin remains controversial. Inhaled heparin (20,000 U daily, 5000 U every 6 h) was safe, but had no effect as prophylactic therapy against nosocomial pneumonia or on recovery from pneumonia in mechanically ventilated patients [139]. However, Olapour et al. (2021) reported that 10,000 U heparin inhaled daily, 5000 U twice a day, for 7 days improved the respiratory and pulmonary status of intubated ARDS patients and reduced the need for mechanical ventilation and days in the ICU [85].

## 10. Inhaled Heparin for Inflammatory Lung Disease in Asthma, Cystic Fibrosis and COPD

### Anti-Viral Effects

Respiratory viruses including rhinovirus (RV), influenza virus, respiratory syncytial (RS) virus, and seasonal human coronavirus, lead to the development of respiratory diseases, including bronchitis, pneumonia, pulmonary fibrosis, and exacerbate the symptoms of bronchial asthma, COPD, bronchiectasis, CF, interstitial pneumonia, and diffuse panbronchiolitis (DPB) through pulmonary inflammation, fibrosis, cell damage, mucus secretion, airway hyperresponsiveness, and secondary bacterial infection (reviewed in [140]).

The production of type I anti-viral cytokines, interferon (IFN)-β and IFN-α from epithelial cells or dendritic cells is lower in patients with asthma and COPD [141,142,143]. Impaired antiviral immune responses are similarly a feature of CF [144]. Human rhinovirus (HRV) is the most common cause of the common cold, and for those with asthma [145], CF [146], or COPD [147], HRV can lead to severe exacerbation of symptoms and potentially fatal complications.

Viral adhesion to HSPG appears to be the first step in the infection process in most cases, followed by interaction of viral proteins with secondary receptors for adhesion and cell entry [90]. Viral proteins binding to HSPG generally contain basic positively charged amino acids that bind to the negatively charged HSPG carbohydrate chain. In most cases, because heparin is more highly sulphated and therefore more negatively charged than HSPG, it blocks viral adhesion to HSPG. The binding of some groups of RV to HSPG is similarly inhibited by soluble heparin [148]. It appears that heparin can displace viruses from the host cell surface at the beginning of the adhesion process, before the viruses have established contact with secondary receptors, which would make them resistant to heparin competition.

Inhaled heparin may therefore have broad-spectrum antiviral effects, through interfering with the binding of viruses to HSPG on airway epithelial cells, and through inhibition of heparanase to reduce viral spread. The ability of unfractionated heparin [90] and a high molecular weight non-anticoagulant heparin [96] to limit viral adhesion to airway epithelial cells, viral spread, and infectivity, supports proposals for the use of inhaled heparin and derivatives to prevent exacerbation of these inflammatory airway diseases.

## 11. Inhaled Heparin in Asthma

The KEGG (Kyoto Encyclopedia of Genes and Genomes) database [149] contains information for the systematic analysis of gene functions, linking genomic information stored in the GENES database with higher order functional information stored in the PATHWAY database. The heparin/HS interactome analysis [16] identified seven heparin binding proteins of the asthma pathway, implicating a role for HBPs in the regulation of asthmatic responses to external stimuli.

Previous reviews considered the mechanisms behind the therapeutic potential of inhaled heparin to treat asthma [150,151]. Pulmonary effects of inhaled heparin may include inhibition of inflammatory cytokine synthesis, and inhibition of chemokine function and the mechanisms leading to inflammatory cell recruitment and activation. Inhibition of neutrophil elastase activity associated with neutrophilic inflammation in severe asthma may be *via* direct inhibitory effects of heparin on the enzyme and/or antioxidant effects that increase the antiprotease shield provided by SLPI and alpha1-antitrypsin. Other activities of heparin include inhibition of complement activation, binding and neutralisation of the tissue-damaging eosinophil basic proteins, as well as disruption of NETS and eosinophil extracellular traps. Consequently, heparin potentially inhibits mucus hypersecretion induced by neutrophil elastase, eosinophil cationic protein, and reactive oxygen species, as well as having mucolytic effects targeting mucin interactions to improve mucus clearance [150]. High levels of HMGB1 in the airways may be a further target for the anti-inflammatory effects of heparin in asthma [152].

Conversely, anti-coagulant effects on fibrin formation in the asthmatic airway [153,154] are likely to be limited by high concentrations of heparin-binding proteins in the inflamed airway that neutralise the anticoagulant activity of heparin (discussed above). Nevertheless, the greatest perceived risk of heparin in the inflamed airways is that of haemoptysis. There is therefore an interest in developing non-anticoagulant derivatives that retain the anti-inflammatory properties of heparin [151,155], such as ODSH [38] (described above), for the management of asthma.

Previous articles have reviewed clinical trials of inhaled unfractionated and LMW heparin in adults and children with asthma and allergy [156,157]. The majority of the studies investigated the effect of inhaled UFH. Inhaled heparin improved lung function in allergen- and exercise-induced asthma, and reduced bronchoconstriction following provocation with water, methacholine, adenosine, histamine, and hypertonic potassium chloride, with no systemic anticoagulation or adverse effects such as haemoptysis.

With respect to clinical trials of inhaled LMW heparin, Ahmed et al. (1999) demonstrated the greater potency of a single dose of LMW heparin, enoxaparin, compared to UFH, for inhibiting exercise-induced bronchoconstriction in patients with asthma [158]. The inhibitory effect of 2 mg/kg dose of enoxaparin was more potent than UFH (80,000 units, 7.5 mg/kg). The post-exercise recovery time was also shorter with enoxaparin. Furthermore, there was no effect of inhaled heparin on systemic anticoagulation measured as anti-Xa activity [158].

In a longer-term study, Fal et al. (2003) reported significant improvement in FEV1 after 14 days treatment with inhaled LMW heparin [159]. Anti-inflammatory effects were also investigated in this study. It was concluded that the mechanisms of action are likely to be prevention of mast cell degranulation, which was reflected in a decrease in serum histamine, decreased recruitment, and activation of eosinophils in the airway (decreased % eosinophils, lower EG2 in BAL fluid), and decreased percentage of lymphocytes in BAL. Interestingly, the effect of LMW heparin was not dose dependent for 5000–10,000 IU delivered twice a day.

Inhaled UFH and LMW heparin are both effective in patients experiencing exacerbation of their asthma. In two patients with acute asthma who had not responded to corticosteroid therapy, 100,000 IU UFH delivered by jet nebuliser improved spirometry and symptoms immediately. Improvements were maximal after 5 days of inhaled therapy, at which point the patients were discharged, and were maintained for 4 weeks [160]. In a recent study in patients with mild–moderate acute asthma not receiving corticosteroids, three doses of LMW heparin (1 mg/kg) delivered every 20 min improved PEFR and FEV1 over 40–60 min of administration [161].

## 12. Inhaled Heparin in COPD

The anti-inflammatory, antioxidant, antinitrosant, mucoregulatory, mucolytic, and tissue repair mechanisms underpinning the potential use of inhaled heparin to treat COPD were previously reviewed [150]. These include inhibition of inflammatory cytokine synthesis, inhibition of chemokine function and the mechanisms leading to neutrophil recruitment and activation in the airways, inhibition of neutrophil elastase activity, antioxidant effects, inhibition of complement activation, disruption of NETS, inhibitory effects on mucus hypersecretion induced by elastase and reactive oxygen species, and mucolytic effects targeting mucin interactions to improve mucus clearance [150]. Additionally, since HMGB1 acting on RAGE and TLR4 represents key pathways for the formation of cigarette-smoke-induced COPD inflammation [162,163], increased pulmonary expression of HMGB1 in COPD is a further potential target for inhaled heparin therapy.

Clinical trials of UFH and LMW heparin in patients with COPD were previously reviewed [150]. All studies reported improvements in pulmonary function irrespective of administration via the subcutaneous, intravenous, or inhaled route, with no effects on systemic coagulation parameters.

In a randomised double-blind study of inhaled UFH in patients with moderate to severe COPD, patients received nebulised inhaled UFH (75,000 or 150,000 IU twice a day) or placebo for 21 days [24]. All patients also received nebulised salbutamol (1 mg) and beclomethasone dipropionate (400 μg) twice daily over the same period. UFH significantly increased FVC following 7 days of treatment with both doses. The higher dose, 150,000 IU b.i.d., significantly increased FEV1 (+249 ± 69 mL compared with placebo) following 7 days of treatment. With both doses of UFH, a clinically significant improvement in exercise capacity and dyspnoea were seen after 21 days of treatment, and there were no serious adverse events or effects on systemic coagulation [24].

More recently, in mechanically ventilated patients with acute exacerbation of COPD, patients were randomly allocated to receive nebulised heparin (25,000 IU) and salbutamol (5 mg) every 6 h, or nebulised salbutamol only (5 mg), for a maximum of 14 days. The co-administration of nebulised heparin with salbutamol significantly increased ventilator-free days compared with salbutamol alone, with no significant effect on coagulation [164].

Thus, inhaled nebulised UFH is safe and provides additional clinical benefit for patients with moderate to very severe COPD, through effects that are likely to be independent of its anticoagulant activity.

## 13. Inhaled Heparin in Cystic Fibrosis

### 13.1. Antimicrobial Effects

Respiratory viruses are common in CF. They are frequently detected during clinical stability and are associated with up to 69% of pulmonary exacerbations [165]. Respiratory infections with respiratory syncytial virus (RSV), rhinovirus (RV), influenza, parainfluenza, and adenovirus are common. Predominant are RVs. Viral infection is associated with decline in pulmonary function and exacerbation in people with CF [166]. Further, viral infection facilitates bacterial colonisation in CF patients [167]. The binding of RSV to CF epithelial cells was shown to promote adherence of *Pseudomonas aeruginosa* in co-cultures, an effect that was blocked by heparin (100 IU/mL) [168]. The antiviral effects of heparin, described above, therefore potentially also have indirect antibacterial effects in CF.

It was reported that UFH is unlikely to have direct antibacterial effects because of its unpredictable inhibition of growth of common respiratory pathogens, including *Pseudomonas aeruginosa* [169]. However, heparin derivatives with low anticoagulant activity, N-acetyl heparin and a glycol-split heparin, (30 mg/kg subcutaneously) reduced bacterial burden in established chronic murine lung infection with CF clinical isolates of *Pseudomonas aeruginosa* [170]. The effect was potentially mediated via competition with heparan sulphate (HS) binding sites for *Pseudomonas aeruginosa* in the lung [171]. Furthermore, the N-acetyl heparin and glycol-split heparin derivatives decreased *Pseudomonas aeruginosa* biofilm formation *in vitro* [170].

The 2-O, 3-O desulphated heparin, ODSH, with low anticoagulant activity has been shown to reduce *Pseudomonas aeruginosa* burden in the lungs of wild-type and CF mice when given *via* subcutaneous or intraperitoneal administration [172,173]. The improvement in bacterial clearance with amelioration of lung injury was mediated by inhibition of HMGB1 binding to TLR2 and TLR4 receptors and restoration of macrophage function [172,173].

Since current evidence suggests that cystic fibrosis transmembrane conductance regulator (CFTR) modulators are unable to eradicate pathogens in patients with established lung disease, and it is unknown whether treatment with CFTR modulators can restore dysfunctional anti-viral responses [174], further evaluation of heparin and heparin derivatives as novel inhaled therapies to reduce infection and inflammation is warranted.

### 13.2. Anti-Inflammatory Effects of Heparin and Derivatives in CF

Within the CF airways, defective ion transport via the epithelial CFTR protein and the associated increase in epithelial sodium channel (ENaC) activity is believed to result in the accumulation of dehydrated, tenacious pulmonary secretions and impaired mucociliary transport. Excessive pulmonary secretions within the lungs stimulate an early sterile neutrophilic inflammatory response, possibly via hypoxic epithelial necrosis and IL-1α signalling [175,176]. Subsequent infection and chronic colonisation by bacterial pathogens stimulate further neutrophilic inflammation, causing progressive proteolytic and oxidative pulmonary damage, resulting in severe bronchiectasis.

**Figure 3:**
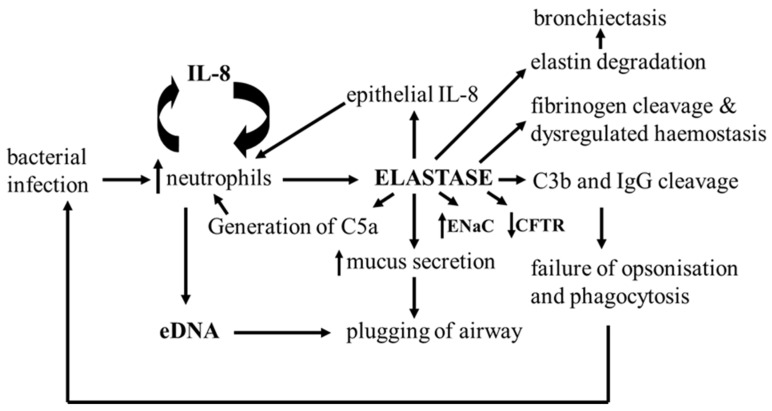
Neutrophil elastase activity is central to the pathogenesis of CF lung disease. Neutrophil elastase is a potent mucus secretagogue and elastolytic tissue enzyme that cripples the immune system and limits airway coagulation, leading to cycles of IL-8-driven infection and inflammation that are difficult to break [175,177,178,179].

Central to the pathogenesis of lung disease in CF are high levels of unopposed neutrophil elastase activity, an enzyme with multiple activities that contribute to defective epithelial ion transport and the self-amplifying cycles of infection and inflammation that characterise the CF airways (Figure 3). Heparin and non-anticoagulant heparin derivatives with anti-elastase and other anti-inflammatory activities have been identified as potential inhaled anti-inflammatory therapies for CF.

Heparanase is an enzyme involved in neutrophil recruitment to the lungs, increased expression of VEGF and angiogenesis, and increased expression of proteases including MMP-9 [180]. Since MMP-9 is highly expressed in CF [181], and is activated by neutrophil elastase activity, heparanase is a further important target for anti-inflammatory therapy with heparin and non-anticoagulant derivatives of heparin [48]. In particular, the high molecular weight non-anticoagulant derivative of heparin, roneparstat, is a stable, potent, and specific inhibitor of heparanase activity [49,182].

The inflammatory response stimulates further mucus production and leads to the accumulation of extracellular DNA (eDNA) from NETs and from necrotic neutrophils, which further increases the viscoelasticity of the airway secretions and correlates with airflow obstruction [183]. We previously showed that the enhanced viscoelasticity of airway secretions in CF is related to secondary infection, decreases with intravenous antibiotic therapy, and correlates with inflammation, measured as NE activity [184]. This process becomes self-perpetuating with amplifying cycles of infection, inflammation, and pulmonary damage that each contribute to the progressive decline in lung function and ultimately result in death.

Unfractionated heparin significantly reduced the elasticity and yield stress, but not the viscosity, of CF sputum *ex vivo*. In addition, heparin enhanced DNase activity in the sputum of patients receiving Pulmozyme therapy. Together, these effects are likely to improve mucus clearance of the airways and reduce airflow obstruction when heparin is inhaled in CF [56].

Pulmonary disease is by far the greatest cause of morbidity and mortality in CF and its effective management is central to adequate patient care. Despite the advent of CFTR modulators, there remains an unmet need for new and more effective anti-inflammatory and mucoactive drugs, as CFTR correction cannot reverse established bronchiectasis, which will continue to predispose people with CF to infection and further airway inflammation, perpetuating the cycle of pulmonary damage [185]. However, in view of the current burden of therapy in patients with CF, new approaches should aim to limit the time spent on treatments. This may be achieved using a single drug such as heparin or a non-anticoagulant derivative with multiple pharmacological actions to improve mucus clearance, limit infection, and reduce local inflammation when inhaled into the airways.

*In vivo* studies showed that heparin derivatives with low anticoagulant activity, N-acetyl heparin, and a glycol-split heparin delivered subcutaneously dampened leukocyte recruitment and cytokine/chemokine production induced by acute and chronic *Pseudomonas aeruginosa* pneumonia in mice [170]. However, these anti-inflammatory effects have not been reported consistently in the few clinical trials of inhaled heparin in people with CF.

#### Clinical Trials of Inhaled Heparin in CF

In a single-dose, dose-ranging study, acute anti-inflammatory effects of inhaled UFH (1000 U/kg, 2000 U/kg) were reported, with inhibition of sputum neutrophil elastase activity at 4 h and inhibition of complement activation at 24 h [186]. The highest dose equated to 10 mg/kg, of which 8% (0.8 mg/kg) was potentially delivered from a jet nebuliser to the airway [187,188], with no systemic anticoagulant effects at any dose [186].

Ledson et al. (2001) investigated the effect of inhaled nebulised heparin, 25,000 IU daily for 7 days, in stable adult CF patients with Burkholderia infection [189]. They reported significantly decreased sputum and serum inflammatory cytokines (IL-6 and IL-8), with subjective sputum mucolysis, but no change in spirometry. All patients tolerated inhaled heparin with no evidence of bleeding, thrombocytopenia, or change in coagulation parameters.

Subsequently, the effect of inhalation of 50,000 IU UFH from a jet nebuliser (250 mg nominal dose, ~20 mg delivered to the airways) twice daily for 2 weeks was investigated in a randomised, double-blind, placebo-controlled crossover study with a 1-week washout period [190]. In this study, heparin inhalation had no significant effect on spirometry, symptoms of sputum clearance, or sputum inflammatory markers, and no effects on systemic coagulation. However, inhaled heparin was deemed safe, indicating that evaluation of larger doses over a longer period was warranted [190].

The variable clinical trial results may reflect the considerable variability in measurements of inflammatory mediators in sputum from CF subjects [191,192], and may have resulted in the different findings in the early uncontrolled studies. Further dose-ranging, placebo-controlled studies are therefore needed.

## 14. Alternative Formulations of Heparin

Studies have shown that heparin can be been reformulated as a dry powder that retains mucolytic effects [193,194].

A phase I/II randomised, placebo-controlled, double-blind trial assessed the safety, tolerability, pharmacodynamics and efficacy of heparin dry powder inhalation, at doses of 11,400 IU [62 mg], 22,800 IU [124 mg], 45,600 IU [248 mg], or placebo, administered twice daily over 4 consecutive weeks in patients with CF [EudraCT Number: 2007-006276-11]. The treatment was well tolerated, without adverse events and with no clinically relevant changes in systemic coagulation parameters. At the two higher doses, clinically relevant improvements in mucus rheology (reduced sputum viscoelasticity) and reduced sputum inflammatory markers (total cell counts, neutrophil elastase activity and IL-6) were reported, with no effect on systemic markers of inflammation or lung function.

The positive results of heparin delivery via the pulmonary route have stimulated research focussed on the preparation and evaluation of heparin in advanced drug delivery systems such as nano/microparticles and liposomes. These formulations are proposed to protect heparin from enzymatic degradation within the airway, achieving long-lasting effects. However, much additional research *in vitro* and *in vivo* is necessary to assess the clinical applicability of this treatment strategy [195].

## 15. Dose-Dependent Effects of Inhaled Heparin on Systemic Anticoagulation

Current pharmaceutical preparations of UFH have an average molecular weight of about 13,000–15,000 Da and specific activity of 180–220 International Units (IU)/mg [196]. The molecular weight and specific activity of commercial preparations of UFH have increased over time, which should be considered when comparing studies of inhaled heparin [197]. The molecular weights of commercial preparations of LMW heparin vary between 2900 and 5000, and the biological properties of LMW heparins are primarily determined by their MW distribution [196]. The ratio of anti-Xa to anti-IIa activity in LMW heparins varies between 1.6 to 4.2, except in bemiparin, which has the lowest MW and an anti-Xa to anti-IIa ratio of 9.6. Dosage of the LMW heparins correlates better with anti-Xa rather than anti-IIa activity, and anti-Xa activity is the only measurement that can be used for monitoring anticoagulant activity. However, because of residual anti-IIa activity, the degree of anticoagulation induced by different LMW heparins may not be comparable at the same anti-Xa plasma concentration. Therefore, monitoring of therapeutic doses of LMW heparin is not routinely required, and LMW heparin may be administered with weight-adjusted dosing in most patients [196].

In 1976, studies in humans showed that inhaled nebulised heparin (162.5 IU/mg) had no effect on systemic coagulation at doses less than 8 mg/kg, which equates to 1300 IU/kg, or 91,000 IU inhaled by a 70 kg average adult. No acute or chronic toxicological effects were reported in any species studied [198].

The dose delivered to the lower respiratory tract (LRT) depends on the nebuliser used. From a jet nebuliser, 8% of the loading dose was delivered to the LRT [187,188]. UFH was inhaled from Sidestream jet nebulisers with nominal doses of 100,000, 200,000, 300,000 or 400,000 International Units (IU) of heparin. Lung function and systemic anticoagulation parameters were measured before and 1, 3, 6, and 24 h after inhalation [188]. The highest LRT dose was 32,000 IU heparin (8% of the loading dose), which did not affect pulmonary function, and had only small non-clinically significant effects on systemic coagulation. The authors concluded that inhaled heparin at these or smaller doses distributes uniformly in the lungs, from where it clears slowly (40% remaining after 24 h), and is safe with respect to pulmonary function and systemic anticoagulation [187,188].

Similarly, Markart et al. (2010) reported that inhalation from 150,000 IU heparin in a jet nebuliser was the threshold dose above which significant effects on systemic coagulation could be detected in healthy subjects [199]. The local alveolar anticoagulant effect was detectable up to 72 h, and the alveolar half-life was estimated at 28 h [199].

However, vibrating mesh nebulisers are more efficient than jet nebulisers for pulmonary drug deposition [200] and are the device of choice in ventilator circuits for delivery of aerosolised UFH in patients with ALI and ARDS [84,135,136,137].

## 16. Potential Side Effects of Inhaled Heparin Therapy

Clinical studies of inhaled heparin in asthma, COPD and CF have consistently reported the safety of inhaled heparin [24,156,157,189,190]. Furthermore, because unfractionated heparin does not cross the bronchial mucosa at doses <8 mg/kg [198], the risk of HIT and other potential side effects such as osteoporosis and alopecia [4] at doses predicted to be clinically useful [24] is low.

Heparin binding proteins are proposed to limit the anticoagulant activity of heparin in the airway. However, despite not being reported, the greatest perceived risk of inhaled heparin in the inflamed airways is that of haemoptysis. Risk of bleeding may be mitigated by the use of non-anticoagulant heparins. The high molecular weight non-anticoagulant heparin, roneparstat, retains many of the pleiotropic functions of unfractionated heparin, and has anti-viral [96] and anti-inflammatory properties, including inhibition of heparanase [94]. Non-anticoagulant derivatives of heparin may therefore provide an effective and safe therapeutic approach when inhaled in the treatment of obstructive inflammatory airway diseases such as asthma, COPD, CF, and non-CF bronchiectasis.

## Data Availability

Data is contained within the article.
